# Pediatrics

**Published:** 1897-08

**Authors:** Maud J. Frye

**Affiliations:** Clinical instructor in diseases of children, Medical Department, University of Buffalo


### PEDIATRICS.

Reported by MAUD J. FRYE, M. D.,
Clinical instructor in diseases of children, Medical Department, University of Buffalo.

### HOSPITALISM IN INFANTS.

HENRY D. CHAPIN, (*Archives of Pediatrics*, May, 1897,) writing after twelve years of hospital experience, says: Owing to their susceptibility to hospitalism infants should be placed in a hospital only under exceptional circumstances, as, for instance, entire inability to secure proper care at home. Only cases of acute illness should be received and these should be discharged immediately on recovery, even if the latter is only partial.

A speedy or satisfactory convalescence is impossible for an infant in a hospital. As a rule, two weeks should be the limit of a young infant's stay. Hospitalism shows itself earlier the younger the infant. One of the first indications is a progressive loss of weight not dependent on the original disease; this may take place when the infant is apparently digesting a suitable artificial food. Accompanying the atrophy there is marked hydremia, dryness of the skin, and wearing off of the hair from the occiput. Hypostatic pneumonia is very apt to develop and, being very insidious, may first be discovered at the autopsy. Female children are apt to develop a vaginitis from the lack of tone of all mucous membranes occurring in hospitalism. Adding to the danger of hospitalism the risks to which the little patients are subjected from outbreaks of contagious diseases, extreme care should be exercised when considering the subject of a hospital for babies. The hygienic conditions must be of the best, the nursing of a very high grade, and a most scrupulous and painstaking oversight exercised. The first signs of hospitalism should be the indication for discharge. If it gets beyond a certain point, no change of food or environment will benefit the infant.

---

### OPIUM IN PEDIATRIC PRACTICE.

IN PRESCRIBING opium (abstract from editorials in *Archives of Pediatrics*, May, 1897,) for both children and adults, its varied and contradictory actions should never be forgotten. These actions may be briefly summarised as follows: (1) Opium stimulates the heart; (2) weakens the respiration by acting upon the respiratory center of the medulla; (3) blunts the sensory nerves; (4) decreases the secretion of the digestive tract, the bronchi, and all mucous surfaces, the liver and various glands; (5) increases, often, the secretion of the skin and kidneys; (6) decreases the action of the unstriped muscle fiber, thus checking peristalsis and spasm of the intestine, spasm of ducts, and action of the bronchi and bladder.

In the diarrheal diseases of children it is contraindicated: (1) in the first stages of acute diarrhea, before the intestinal canal has been freed from decomposing matter; (2) when the passages are infrequent and of bad odor; (3) when there is a high temperature or cerebral symptoms are present; (4) when its use is followed by elevation of temperature or the passages become more offensive—symptoms which indicate toxic infection from putrefying intestinal contents.

It is indicated in diarrheal diseases: (1) when the passages are frequent, with pain; (2) when the passages are large and watery; (3) in dysenteric diarrhea, together with castor oil or a saline; (4) in late stages, with small, frequent, nagging passages; (5) when the passages consist largely of undigested food, and the bowels act as soon as food is taken into the stomach, the dose should be as small as possible, sufficient being given to relieve pain and check peristalsis. It should be given alone, at intervals, sufficient to allow the effect of one dose to partially subside before another is given. In respiratory diseases, in general terms, it may be said that the more deeply the respiratory tract is involved, the more cautiously should opium be administered. In the dry, painful cough of pleurisy it may be given more freely than in almost any other condition. In the early, dry stage of bronchitis it may be given in moderate doses, as well as for the harassing cough of the later stages, if the respiration is free and the circulation is good. One of the most efficient means of relieving spasmodic croup is a full dose of opium combined with ipecac.

---

### THERAPEUTIC VALUE OF A SUDDEN CHANGE OF DIET.

Angel Money (*Pediatrics*, May 15, 1897,) writes editorially on this topic. He says: The class of disease in which this mode of therapeutics finds most application is usually chronic, frequently neurotic, and not uncommonly affections of the mucous membranes. Thread-worms are often successfully treated by a complete reversal in the mode of feeding. Sometimes a chronic bronchial catarrh will yield to a similarly directed treatment. Asthmatic attacks are sometimes made to alter their rhythm and occasionally to cease altogether for long periods. Cases of nocturnal incontinence and enurises may be checked by a profound alteration of the dietary. The sudden withdrawal of all saccharine and starchy stuffs from the food and its replacement by pure proteids with salts, extractives and water is a very good mode of treating a dilated stomach or flatulent dyspepsia without much distension of this organ. Sweetbreads, lambs' trotters, tripe, calf's head and feet, isinglass and gelatine blanc-mange, unsweetened jellies, vegetable marrow, well-cooked and well-drained, cucumbers, similarly treated and freed from skins and seeds, lettuce, thoroughly boiled and freed from all excess of moisture, very young green peas, very tender French beans—all these may be utilised. If the dietetic treatment is not to last longer than a week, gluten bread, almond biscuits and soya nuts make other provisions comparatively free from fermenting factors. Most of the therapeutic value of a complete change of diet is to be obtained by its adoption for short periods only.

---

### THE CHEMICAL EXAMINATION OF HUMAN BREAST MILK.

ADRIANCE (Vanderpool and John S., *Archives of Pediatrics*, February, 1897,) summarise as follows, as the result of the examination of 200 cases. To secure healthy milk, a mother should have: (1) A good constitution; (2) healthy environment; (3) simple, nutritious diet with enough albuminous food; (4) regular exercise in the open air.

(1) Excessive fats or proteids may cause gastro-intestinal symptoms in the nursing infant; (2) excessive fats may be reduced by diminishing the nitrogenous elements in the mother's diet; (3) excessive proteids may be reduced by the proper amount of exercise; (4) excessive proteids are especially apt to cause gastro-intestinal symptoms during the colostrum period; (5) the proteids, being higher during the colostrum period of premature confinement, present dangers to the untimely-born infant; (6) deterioration in human milk is marked by a reduction in the proteids and total solids, or in the proteids alone; (7) this deterioration takes place normally during the later months of lactation, and unless proper additions are made to the infant's diet, is accompanied by a loss of weight or a gain below the normal standard; (8) when this deterioration occurs earlier, it may be the forerunner of the cessation of lactation, or well-directed treatment may improve the condition of the milk.

